# Determinant factors in the use of modern contraception in urban and rural areas in Western Indonesia

**DOI:** 10.1186/s12889-025-23299-7

**Published:** 2025-06-02

**Authors:** Qorinah Estiningtyas Sakilah Adnani, Yuninda Loviana Ersianti, Siti Khuzaiyah, Kadar Ramadhan, Ari Indra Susanti, Rikke Damkjær Maimburg, Kughong Reuben Chia

**Affiliations:** 1 Department of Public Health, Faculty of Medicine, Universitas Padjadjaran, Bandung, Indonesia; 2 Master of Midwifery Program, Faculty of Medicine, Universitas Padjadjaran, Bandung, Indonesia; 3 Midwifery Program, Faculty of Health Sciences, Universitas Muhammadiyah Pekajangan, Central Java, Pekalongan, Indonesia; 4 Nursing and Midwifery Program, PAPRSB Institute of Health Sciences, Universiti Brunei Darussalam, Bandar Seri Begawan, Brunei Darussalam; 5 Department of Midwifery Poltekkes Kemenkes Palu, Palu, Indonesia; 6 Department of Clinical Medicine, Aarhus University, Aarhus, Denmark; 7 Department of Midwifery, University College of Northern Denmark, Aalborg, Denmark; 8 Doctor of Medicine, Faculty of Health Sciences, University of Bamenda, Bambili, NW Region Kamerun

**Keywords:** Contraception, Factors, Married women, Family planning, Western Indonesia

## Abstract

**Background:**

Family planning remains essential to ensure women’s autonomy, health, and maternal health outcomes and safeguard population expansion. Knowledge is needed to understand how modern contraceptives are used in both urban and rural areas in Indonesia. The objective of this study was to analyze the utilisation of modern contraceptives between rural and urban areas in Western Indonesia as well as associated factors.

**Methods:**

A cross-sectional study using secondary data from the nationally representative 2017 Indonesian Demographic and Health Survey (IDHS). Bivariate and multiple logistic regression were conducted to analyse the data.

**Results:**

Twelve thousand eight hundred thirty-one married women aged 15 to 49 became the sample of this study, with 6,955 (54.2%) residing in urban areas and 5,876 (45.8%) in rural areas, utilising current contraceptive methods. The research revealed that from the users-only data, 86.3% of married women in urban areas and 92.2% in rural areas used modern contraception. Rural married women aged 20–44 exhibited greater odds of utilising modern contraception compared to their urban counterparts. Married women aged 15–24 in rural areas had 4.0 times higher odds of using modern contraception than their urban counterparts. Married women with no education had higher odds of using modern contraceptives in both urban (4.8 times) and rural areas (3.9 times). In urban areas, those with 1–3 children had 5.9 times higher odds compared to women with ≥ 7 children, while in rural areas, women with no children had 5.0 times higher odds. Urban women in the second wealth index had 1.2 times higher odds compared to those in the lowest, and in rural areas, women in the highest wealth index had 1.9 times higher odds.

**Conclusion:**

Modern contraceptive use is similar in urban and rural areas of Western Indonesia, with sociodemographic factors significantly influencing use. Key differences include the impact of education, number of children, and wealth index, with lower use among women with secondary education and those in the lowest wealth group. Family planning efforts should target education gaps, promote early family planning, and expand access to services for low-income women, while also strengthening outreach to wealthier urban women.

## Introduction

Less than half of the need for family planning is met by modern contraceptive methods in 43 countries, 32 of which are low-income countries [1]. In Indonesia, a continuous rise in the population has been experienced during the last five years. In 2017, the population included 264,498,852 citizens, which increased by approximately 4.5% o 273,753,191 in 2021 [2]. The maternal mortality ratio (MMR) in Indonesia remained persistently high in 2020, reaching 189 per 100,000 live births [3]. Aryanty’s study in 2021 revealed that the MMR correlates with several factors, including an increased number of births (four or more), a higher concentration of impoverished households, reduced density of hospitals, a heightened presence of traditional birth attendants (TBAs), residing outside the Java-Bali region, and diminished prevalence of contraceptive use [4].

To accelerate the reduction of preventable maternal deaths, it is crucial to promote contraceptive utilisation and diminish unmet family planning needs [5, 6]. The prevalence of contraceptive use contributed to a significant reduction in maternal mortality, with the Utomo study showing a 37.5–43.1% reduction between 1970 and 2017 [4, 7]. In addition, data from DHS Indonesia from 2002 to 2017 shows that the Family Planning Program has been stagnant, with approximately 63.6% of people using any method of contraception and 57.2% using modern methods in 2017 [8]. The National Population and Family Planning Board’s strategic plan for 2020–2024 aims to elevate the modern contraceptive prevalence rate (CPR) from 61.8% in 2020 to 63.4% in 2024, concurrently aiming to decrease the unmet need for family planning from 8.6% in 2020 to 7.4% in 2024 [9].

Contraceptive methods are often divided into two types: traditional and modern. Conventional methods include withdrawal and rhythm methods and are considered less effective than modern contraceptive methods, including sterilisation, Intrauterine devices (IUD), implants, injections, and oral pills [10]. Theory-based interventions such as the Social Determinants of Health Theory (SDH), Health Belief Model (HBM), Social Cognitive Theory (SCT), and Transtheoretical Model are often used to guide contraceptive behavior change [11, 12]. According to the Health Belief Model, various dimensions associated with contraceptive use include demographic, social, structural, psychological, and reproductive factors [13]. Furthermore, the Social Determinants of Health (SDoH) framework is suitable for this study to understand how factors such as socioeconomic status, income, education, and health literacy play a key role in shaping health behaviors related to contraceptive use [12, 14].

Jamali’s study using data from the Pakistan Demographic and Health Survey (PDHS) shows that 27.7% of sexually active married women use modern contraceptives, influencing factor women who being less use modern contraceptives include women from Balochistan, younger age groups, less educated, the poorest, those not visited by fieldworkers, and those with fewer than five children [15]. Simon’s study, based on the 2016/17 Haiti Demographic and Health Survey (HDHS), reveals that condom use was more common among sexually active individuals (15.4%; 95% CI: 14.0–16.8%), particularly among teenagers, urban residents, those with higher levels of education, wealthier households, knowledge of the ovulatory cycle, fewer lifetime sexual partners, and those with non-spousal partners, such as boyfriends or casual acquaintances [16]. On the other hand, five obstacles have been identified that prevent multiparous Indonesian women from using contraceptives, including being urban residents, younger, uneducated, unemployed, and having a low socioeconomic status [17]. Several previous studies highlight that regional location can significantly influence married women’s access to modern contraceptives.

Indonesia categorizes places of residence into two types: urban and rural. Living in one of the specific areas may influence the preference for contraceptive methods. Previous studies have indicated that individuals in the Java-Bali, Sumatra, and Kalimantan regions are more likely to use hospital services than those in Papua and Maluku, likely due to the stronger healthcare infrastructure and easier access in these areas [18]. The research demonstrated that urban areas in Indonesia had higher healthcare utilization than rural districts. This disparity was primarily attributed to geographical inequalities, exacerbated by distance, expense, and a lack of services in rural regions [19, 20]. Research in Indonesia revealed that the majority of contraceptive users were urban women (26,197; 63.4%) [21]. This study investigated contraceptive use only among women who were practicing contraception. Therefore, further analysis is needed to complement the data on modern contraceptive use in Indonesia.

Although studies on modern contraception among married women in Indonesia exist, they only focus on the 2017 IDHS in urban areas and cover the country as a whole [4, 22, 23]. Currently, there is limited detailed analysis of modern contraceptive use categorised by urban and rural areas in the 2017 IDHS data, particularly focused on the western region of Indonesia. This study aimed to analyse the differences in modern contraceptive use between rural and urban areas in the western part of Indonesia, specifically focusing on the regions of Sumatra, Java, and Kalimantan. Understanding associated factors with modern contraceptives may improve our knowledge of how to plan suitable programs for the community to enhance the utilisation of modern contraceptives in urban and rural areas in Indonesia.

## Materials and methods

### Design and data source

This cross-sectional study comprised a secondary analysis of nationally collected data in 2017 from the Indonesian Demographic and Health Survey (IDHS). The Indonesian Demographic and Health Survey (IDHS), conducted every five years, is part of the global Demographic and Health Survey (DHS) program and is carried out by Statistics Indonesia (BPS) in collaboration with the National Population and Family Planning Agency (BKKBN) and the Ministry of Health. The survey employs international standardised questionnaires and rigorous data collection techniques. All women aged 15 and 49 were eligible to participate in the study. Trained fieldworkers conducted face-to-face interviews with married women. Two questionnaires were used to collect information from the married women and one from their households, respectively.

The survey design employed a two-stage stratified cluster sampling method to ensure the data’s representativeness. Several census districts were selected in the first sampling stage using a technique based on probability proportional to size. From the inventory of census blocks, a random sample of ordinary households was selected in the second sampling stage. (9). With a response rate of 98% at the household level, the IDHS provides comprehensive data and current estimates of fundamental demographic and health indicators. Data that was incomplete or unavailable was omitted from the database.

The Inner-City Fund (ICF) International authorized the use of the data for scientific research. The ICF Office of Client Resources (OCR) Macro (number 45 CFR 46) and the National Board Review of the Ministry of Health, Republic of Indonesia, granted ethical approval for the IDHS, and all participants provided informed consent before the study. Data could be accessed from https://dhsprogram.com/data/available-datasets.cfm by submitting an online request to the ICF. The authors had no privileged access to these data.

The object of this study was married women aged 15–49 years who participated in the 2017 Indonesian Demographic and Health Survey (IDHS) and were using contraceptive methods, either modern or traditional. The study analysed data from 49,627 women to generate findings that are representative of Indonesia as a whole and its 34 provinces, offering a comprehensive understanding of contraceptive use across the country. The sample was collected from respondents who provided relevant data on their contraceptive use during the survey. The western part of Indonesia was selected due to its diverse population and socio-economic conditions, offering a valuable perspective on regional disparities in contraceptive use. This region includes densely populated and economically significant areas like Sumatra, Java, and Kalimantan, making it essential for understanding how factors like healthcare access and infrastructure influence family planning in both urban and rural settings.

### Variables

The dependent variable in this study was the current contraceptive utilisation among married women aged 15–49. Contraceptive methods in this study were classified as “traditional” or “modern”. Traditional contraceptive methods include: periodic abstinence (rhythm and calendar method), withdrawal (coitus interruptus), or country-specific traditional methods of proven effectiveness, and folk methods, including locally described methods and spiritual methods of unproven effectiveness, such as herbs, amulets, gris-gris, etc. Modern contraceptive methods include; female sterilisation, male sterilisation, oral contraceptive (pill), intrauterine contraceptive devices (IUD), injectables, implants, male condoms, female condoms, diaphragm, lactation amenorrhea method (LAM), standard days method (SDM), emergency contraception, or country-specific modern methods and other modern contraceptive methods respondent mentioned (including cervical cap, contraceptive sponge, and others) [24].

There are other considerations regarding contraception, such as breastfeeding, protracted breastfeeding, and prolonged abstinence, which are not considered contraceptive methods. The lactational amenorrhea method relies on three factors: Since the last birth, the woman has been amenorrheic; the last birth occurred within six months; the woman is exclusively or predominantly lactating. Only the following are used in the DHS description of LAM: Women who use a method requiring frequent lactation throughout the day and night for up to six months after childbirth until their menstrual cycle returns. This description differs from the official LAM criteria by omitting exclusive or predominant breastfeeding (which is based on whether or not the child received complementary liquids and foods), substituting frequency of breastfeeding (frequent night-time feeding is not a requirement), and omitting the criterion that the woman is aware that another method of contraception is required. Therefore, the DHS description may include women who respond affirmatively despite having never heard of the term LAM or programs that teach the method, thereby overestimating knowledge and use [24].

There were 11 independent variables in this study, including residence, maternal age, education and occupation, partner’s education, source of gaining information on family planning (FP) (radio, TV, newspaper, or the internet), number of children, desire to use a contraceptive, and wealth index. Residence was categorised as “rural” and “urban”. Maternal age was classified as 15–19, 20–24, 25–29, 30–34, 35–39, 40–44, and 45–49. Married women and partner education were classified as “no education”, “primary education”, “secondary education”, and “higher education”. Occupation was classified as “not working” and “working”. Information sources regarding family planning (FP) were categorised as “no” and “yes”. The number of children was classified as “no child”, “1–3”, “4–6”, and “≥7”. Desire to use contraception was categorised as “both want” if both married women and their husbands want to use modern contraceptives, “husband wants more” if the husbands have higher intention to use modern contraceptives for their wife, “husband wants less” if the husbands have less intention for his wife to use a modern contraceptive, “don’t know” if the married women don’t know about their husband’s desire. The last variable was the wealth index, categorised as “lowest”, “second”, “middle”, “fourth”, and “highest”.

### Statistical analysis

Distributions of the sociodemographic characteristics, including maternal age, education, occupation, partner education, sources of information about family planning, number of children, desire, wealth index, and current contraceptive, were calculated using descriptive statistics. Binary logistic regression was used to examine the association between each exposure and the outcome variable, and we reported crude odds ratio (OR), 95% confidence interval (CI), and p-values. The final multivariable logistic regression model comprised independent variables that were significant at the bivariable level (p-value < 0.001). Multiple logistic regression analysis was performed to analyse modern contraceptive utilisation among married women in urban and rural areas. The odds ratio (OR) with a 95% confidence interval (CI) presents the association between the variables. All reported *p*-values are two-sided, and the level of statistical significance was 5%. Statistical analysis was performed using STATA 16 (STATA-Corp, College Station, TX, USA, 2020).

## Results

The research population consisted of all married women aged 15 to 49 with children younger than five years old (0–59 months). A total of 12,831 married women aged 15 to 49 who were contracepting, with 6,955 (54.2%) from urban areas and 5,876 (45.8%) from rural areas.

### Respondent characteristics

Table 1 presents the distribution of maternal age, education, occupation, partner education, sources of information about family planning, number of children, desire to use contraception, wealth index, and current contraception usage. The highest likelihood of respondents in urban and rural areas falls in the 35–39 age category. In urban areas, 23.5% of respondents fall into this category, while in rural areas, it is 21.9%. The lowest likelihood of respondents in both areas falls in the 15–19 age category, with only 1.0% in urban areas and 0.2% in rural areas. The highest likelihood of respondents with secondary education falls in urban areas at 60.7%, while in rural areas, it is 46.7%. Most responders in urban and rural areas were working, with 58.9% in urban areas and 58.7% in rural areas. The highest likelihood of respondents’ education and partners with secondary education was felt in urban areas at 61.0%, while in rural areas, it is 47.0%.

Regarding exposure to family planning (FP) messages, a higher likelihood of urban married women had heard about FP on TV (63.7%) and radio (9.5%) compared to rural married women (TV: 56.6%; radio: 8.1%). Similarly, a higher likelihood of urban married women had information about FP in newspapers (14.3%) and through the Internet (23.5%) compared to rural married women (newspapers: 8.3%; Internet: 10.0%).

Most responders in urban and rural areas had 1–3 children, with 89.0% in urban areas and 86.9% in rural areas. The highest likelihood of respondents in urban and rural areas wanted to have the same number of children as their husbands, with 67.0% in urban areas and 69.9% in rural areas.

Regarding the wealth index, more urban married women belonged to the highest (33.2%) and more affluent (27.9%) wealth quintiles. A higher proportion of rural married women, in comparison, belonged to the second (27.9%) wealth quintiles.

The likelihood of married women currently using modern contraceptive methods was higher in rural areas (92.2%) compared to urban areas (86.3%), while the likelihood of married women using traditional contraceptive methods was lower in rural areas (7.8%) (13.7%).

**Table 1:** Percentage distribution of study sample in urban and rural areas by background characteristics: Western Indonesia DHS 2017

	Urban		Rural	
	*n*	%	*n*	%
**Maternal Age**				
15–19	68	1.0	102	0.2
20–24	503	7.7	595	10.5
25–29	1,046	15.0	973	16.6
30–34	1,418	20.1	1,207	19.7
35–39	1,614	23.5	1,299	21.9
40–44	1,388	19.5	1,044	17.4
45–49	918	13.3	656	12.1
**Maternal education**				
No education	46	0.7	79	1.1
Primary	1,603	24.5	2,551	46.3
Secondary	4,229	60.7	2,827	46.7
Higher	1,077	14.1	419	6.0
**Maternal occupation**				
Not working	2,814	41.0	2,306	41.3
Working	4,141	59.0	3,570	58.7
**Partner´s education**				
No education	48	0.7	86	1.5
Primary	1,511	23.2	2,558	46.2
Secondary	4,288	61.0	2,896	47.0
Higher	1,108	15.1	336	5.3
**FP information on Radio**				
No	6,277	90.5	5,413	92.0
Yes	678	9.5	463	8.1
**FP information on TV**				
No	2,574	36.3	2,662	43.4
Yes	4,381	63.7	3,214	56.6
**FP information from the newspaper**				
No	5,895	85.7	5,407	91.7
Yes	1,060	14.3	469	8.3
**FP information from the internet**				
No	5,256	76.5	5,325	90.1
Yes	1,699	23.5	551	10.0
**Number of children**				
0	22	0.4	32	0.8
1–3	6,103	89.0	4,974	86.9
4–6	799	10.2	810	11.5
≥ 7	31	0.4	60	0.9
**Desire**				
Both want	4,589	67.0	4,047	69.9
Husband wants more	1,450	20.6	995	16.8
Husband wants less	408	5.6	273	4.6
Don’t know	508	6.8	561	8.7
**Wealth Index**				
Lowest	370	5.2	1,508	23.2
Second	920	13.3	1,671	27.9
Middle	1,451	20.5	1,340	23.8
Fourth	1,937	27.9	933	16.9
Highest	2,277	33.2	424	8.2
**Current Contraceptive Method**				
Traditional	1,060	13.7	505	7.8
Modern	5,895	86.3	5,371	92.2
**Total**	**6**,**955**	54.2	**5**,**876**	45.8

### Bivariate analysis

Table 2 presents the bivariate analysis results between predictor variables related to modern contraceptive utilisation in rural and urban areas.

The findings of the bivariate analysis indicate a significant association between socioeconomic factors and modern contraceptive use. Variables such as age, education level, partner’s education, and exposure to family planning information via the internet (*p* < 0.001) were significantly associated with modern contraceptive use in both rural and urban areas. A notable distinction was observed in urban areas, where exposure to family planning information through newspapers and the wealth index (*p* < 0.001) showed a significant association, unlike in rural areas.

Women’s age in both rural and urban settings was significantly associated with modern contraceptive use (*p* < 0.001). In urban areas, younger women had the highest percentage of modern contraceptive use, whereas in rural areas, the percentage remained relatively stable across all age groups. Education level also showed a significant association with modern contraceptive use in both settings (*p* < 0.001). Interestingly, women with primary or no education had the highest proportion of modern contraceptive use in both urban and rural areas, compared to women with higher levels of education. On the other hand, women’s occupational status showed no significant association with modern contraceptive use in either setting. Nevertheless, the percentage of non-working women who used modern contraceptives was slightly higher than that of working women in both areas.

The education level of women’s partners in both rural and urban areas was significantly associated with modern contraceptive use (*p* < 0.001). In both areas, women whose partners had no education had the highest percentage of modern contraceptive use compared to those whose partners had higher levels of education. Thus, the lowest level of partner education was associated with the highest use of modern contraceptives in both urban and rural areas.

Women who heard about family planning (FP) on the radio showed no significant association with modern contraceptive use in either urban or rural areas. In both areas, the percentage of women who had not heard FP messages on the radio was higher than those who had. Similarly, exposure to FP messages on television was not significantly associated with modern contraceptive use in urban or rural areas. In urban areas, the percentage of women who heard FP messages on TV was slightly higher than those who did not, while in rural areas, the percentages were relatively similar.

Women who heard about FP in newspapers showed a significant association with modern contraceptive use in urban areas (*p* < 0.001), but not in rural areas. In both areas, the percentage of women who had not heard FP messages in newspapers was higher than those who had. Exposure to FP messages on the internet was significantly associated with modern contraceptive use in both urban and rural areas (*p* < 0.001). In both areas, the percentage of women who had not accessed FP messages on the internet was higher than those who had.

The number of children among married women showed no significant association with modern contraceptive use in either area. In urban areas, women with 1–3 children had a higher percentage of modern contraceptive use compared to their counterparts, while in rural areas, women with no children had the highest percentage of modern contraceptive use. The desire to use modern contraception also showed no significant association in either area. In both areas, women who answered “don’t know” regarding their desire to use modern contraception had a higher percentage of use compared to their counterparts. Meanwhile, the wealth index showed a significant association with modern contraceptive use in urban areas (*p* < 0.001), but not in rural areas. Interestingly, both areas shared a pattern in which women in the lowest wealth category had a higher percentage of modern contraceptive use compared to their counterparts.

**Table 2:** Percentage distribution of contraceptive method used in urban and rural areas and the *p*-value of the bivariate association by background characteristics: Western Indonesia DHS 2017

Variables	Urban			*p*-value	Rural			*p*-value
	*N* (6955)	Traditional (%) (*n* = 1060)	Modern (%) (*n* = 5895)		*N* (5876)	Traditional (%) (*n* = 505)	Modern (%) (*n* = 5371)	
**Age**								
15–19	68	3.0	97.1	< 0.001	102	3.7	96.3	< 0.001
20–24	503	8.6	91.4		595	6.3	93.7	
25–29	1,046	12.9	87.1		973	6.7	93.3	
30–34	1,418	11.6	88.4		1,207	7.2	92.8	
35–39	1,614	12.5	87.5		1,299	6.5	93.5	
40–44	1,388	14.7	85.3		1,044	8.5	91.5	
45–49	918	22.1	77.9		656	13.3	86.7	
**Education**								
No education	46	4.1	95.9	< 0.001	79	7.8	92.2	< 0.001
Primary	1,603	8.8	91.2		2,551	4.8	95.2	
Secondary	4,229	13.0	87.0		2,827	9.4	90.6	
Higher	1,077	25.5	74.6		419	18.1	81.9	
**Occupation**								
Not working	2,814	12.3	87.7	0.008	2,306	6.7	93.3	0.012
Working	4,141	14.7	85.3		3,570	8.6	91.4	
**Partner of Education**								
No education	48	0.5	99.6	< 0.001	86	6.4	93.6	< 0.001
Primary	1,511	8.7	91.3		2,558	5.1	94.9	
Secondary	4,288	12.6	87.4		2,896	9.4	90.6	
Higher	1,108	26.2	73.8		336	17.2	82.8	
**I heard FP on the Radio.**								
No	6,277	13.4	86.6	0.052	5,413	7.7	92.3	0.385
Yes	678	16.3	83.7		463	8.9	91.2	
**I heard FP on TV**								
No	2,574	12.1	87.9	0.013	2,662	7.6	92.4	0.673
Yes	4,381	14.6	85.4		3,214	7.9	92.1	
I heard about FP in the newspaper.								
No	5,895	12.8	87.2	< 0.001	5,407	7.6	92.4	0.132
Yes	1,060	18.9	81.1		469	9.8	90.2	
**Internet**								
No	5,256	12.0	88.0	< 0.001	5,325	7.4	92.6	< 0.001
Yes	1,699	19.2	80.8		551	11.7	88.3	
**Number of children**								
0	22	24.0	76.0	0.001	32	2.4	97.6	0.001
1–3	6,103	13.2	86.8		4,974	7.4	92.6	
4–6	799	16.7	83.3		810	10.7	89.3	
≥ 7	31	39.3	60.7		60	17.9	82.1	
**Desire**								
Both want	4,589	13.5	86.5	0.018	4,047	7.5	92.5	0.119
Husband wants more	1,450	14.8	85.2		995	9.6	90.4	
Husband wants fewer	408	16.9	83.1		273	8.8	91.2	
Don’t know	508	9.7	90.3		561	6.2	93.8	
**Wealth Index**								
Lowest	370	8.3	91.7	< 0.001	1,508	6.8	93.2	0.069
Second	920	7.8	92.2		1,671	7.1	92.9	
Middle	1,451	11.7	88.3		1,340	8.0	92.0	
Fourth	1,937	12.6	87.5		933	8.6	91.4	
Highest	2,277	19.1	81.0		424	10.8	89.2	

### Multivariate analysis

Following the execution of bivariate analysis, the researchers incorporated variables with a *p*-value < 0.2 into the multivariate analysis, as shown in Table 3. P-value < 0.2 was used for initial variable selection, as it is commonly applied to retain potential confounders by allowing a 20% level of confounding and ensuring relevant variables are considered for inclusion in the final regression model [25, 26]. All variables were incorporated for multivariate analysis in the urban group. In the case of the rural group, the variables ‘heard information on radio and TV’ were removed from the multivariate analysis. At this level, multiple logistic regression was utilized.

The analysis reveals that the older married women are, the lower their use of modern contraceptives in both urban and rural areas. Married women aged 15–24 in rural areas had 4.0 times higher odds of using modern contraceptives compared to those aged 45–49, while in urban areas, married women aged 15–24 had 3.4 times higher odds compared to their counterparts aged 45–49. Meanwhile, married women aged 40–44 had the lowest odds of using modern contraceptives compared to those aged 45–49, with odds of 1.7 times in urban areas and 1.8 times in rural areas.

The higher the education level of married women, the lower the probability of using modern contraceptives in both urban and rural areas. In urban areas, married women with no education had 4.8 times higher odds of using modern contraceptives compared to those with higher education. In rural areas, married women with primary education had 3.9 times higher odds of using modern contraceptives compared to those with higher education. On the other hand, women with secondary education had the lowest odds of using modern contraceptives, with odds of 1.4 times in urban areas and 1.7 times in rural areas compared to those with higher education.

Married women who have a partner with primary education in both areas have a higher probability of using modern contraceptives compared to those with higher partner education. In urban areas, married women who have a partner with primary education have 2.1 times higher odds of using modern contraceptives compared to those with higher partner education, while in rural areas married women who have a partner with primary education have 2.4 times higher odds of using modern contraceptives compared to those with higher partner education. Married women who have not heard about FP on the internet in urban areas had 1.4 times higher odds of using modern contraceptives compared to those who had heard about it, while in rural areas married women who have not heard about FP on the internet had 1.7 times higher odds of using modern contraceptives compared to those who had.

Married women who have 1–3 children in urban areas had 5.9 times higher odds of using modern contraceptives compared to those with ≥ 7 children, while in rural areas, married women with 0 children had 5.0 times higher odds of using modern contraceptives compared to those with ≥ 7 children. In urban areas, married women categorized in the second wealth index had 1.2 times higher odds of using modern contraceptives compared to married women in the lowest wealth index, while in rural areas married women categorized in the highest wealth index had 1.9 times higher odds of using modern contraceptives compared to married women in the lowest wealth index.

**Table 3:** Adjusted odds ratio (AOR), *p*-value, and 95% AOR confidence interval (CI) of the binary logistic regression model of determinants of modern contraceptive use: Western Indonesia DHS 2017

Variables	Urban				Rural			
	AOR	*p*-value	95%CI		AOR	*p*-value	95%CI	
			lower	upper			lower	upper
**Age**								
15–24	3.4	< 0.001	2.3	5.0	4.0	< 0.001	2.6	6.1
25–29	2.2	< 0.001	1.7	3.0	3.5	< 0.001	2.4	5.1
30–34	2.5	< 0.001	1.8	3.4	3.0	< 0.001	2.1	4.1
35–39	2.2	< 0.001	1.8	2.8	2.7	< 0.001	1.9	4.0
40–44	1.7	< 0.001	1.4	2.2	1.8	< 0.001	1.3	2.6
45–49	ref				ref			
**Education**								
No education	4.8	0.036	1.1	21.0	3.0	0.022	1.2	7.7
Primary	2.0	< 0.001	1.4	2.8	3.9	< 0.001	2.6	5.8
Secondary	1.4	0.004	1.1	1.8	1.7	0.001	1.2	2.4
Higher					ref			
**Partner of Education**								
Primary	2.1	< 0.001	1.5	2.9	2.4	< 0.001	1.5	3.9
Secondary	1.6	< 0.001	1.3	2.1	1.4	0.087	0.9	2.1
Higher	ref				ref			
**Internet**								
No	1.4	0.002	1.1	1.7	1.3	0.104	0.9	1.8
Yes	ref				ref			
**Number of children**								
0	1.5	0.518	0.4	5.4	5.0	0.188	0.4	55.8
1–3	5.9	< 0.001	2.6	13.2	2.3	0.051	1.0	5.3
4–6	4.1	0.001	1.8	9.0	1.7	0.243	0.7	3.9
≥ 7	ref				ref			
**Wealth Index**								
Lowest	ref				ref			
Second	1.2	0.457	0.7	1.9	1.2	0.251	0.9	1.6
Middle	0.9	0.548	0.5	1.4	1.2	0.308	0.9	1.7
Fourth	0.9	0.704	0.6	1.5	1.5	0.037	1.0	2.2
Highest	0.9	0.629	0.5	1.5	1.9	0.005	1.2	3.0

## Discussion

The proportion of married women currently using modern contraceptives was higher in rural areas (92.2%) than in urban areas (86.3%). The bivariate analysis demonstrates that the rate of modern contraceptive use in urban and rural areas remains consistently similar, with no significant differences observed. Nevertheless, this analysis revealed that married women’s age, education level, partner’s education, wealth index, and exposure to family planning information via the internet and newspapers were significantly associated with modern contraceptive use. The distinction between the two settings lies in the differing impacts of family planning information accessed through newspapers and the wealth index, which highlights contextual variations in the factors influencing contraceptive choices.

This study found that modern contraceptive use remains lower in urban settings (86.3%) compared to rural settings (92.2%). Consistent with this study, the Zambia Demographic and Health Survey (ZDHS) found higher use of modern contraceptives among adolescents in rural areas (13.7%) than in urban areas (9.8%) [27]. This finding is different from previous studies, which showed that women in urban areas tend to use contraception three times more frequently than women residing in rural regions of Congo [28]. The reason for the higher use of modern contraception in rural areas is that social networks and communal interactions are often strong. When modern contraception becomes normalized within a community through the KB Village Program—whether through peer influence, community leaders, or healthcare workers—it can encourage others to adopt similar behaviors [29].

Maternal age was strongly associated with the use of modern contraceptives. Urban and rural areas showed that younger women are more likely to use modern contraceptives compared to older women. Married women aged 15–24 had 4.0 times higher odds of using modern contraceptives in rural areas and 3.4 times higher odds in urban areas. Another study in Zambia used ZDHS, focusing on adolescent girls aged 15–19 years, and found that modern contraceptive use was higher in rural regions than in urban areas, at 13.7% versus 9.8% [27]. The result of multivariate analysis showed that married women aged 15–19 in urban areas had 11.2 times higher odds of using modern contraceptives. This study supported a previous study among married women in Liberia aged 20–24 years (AOR = 2.08) and 25–29 years (AOR = 1.73) who were more likely to use modern contraceptives than those aged 45–49 years [30].

As of 2019, 10.82% of teenagers in Indonesia were engaged in early marriages, and the nation maintains a high prevalence of child marriage that surpasses the national objective [31, 32]. The correlation between contraceptive utilization and fertility in adolescents is affected by variables including the age of initial sexual activity and marital status [33]. One reason for the higher use of modern contraception among young women is that young married women often wish to delay pregnancy [34]. On the other hand, older adults were less likely to engage with technology than the younger population [35]. Having a social, provider, or combined network was positively associated with increased contraceptive use compared to having no family planning network [36].

In general, the results of this study showed that the intention to use contraception decreases as women age among women aged 15–49. In line with a study in Ethiopia showing that women in the age group of 20–24 years old (aOR:1.66), 25–29 (aOR:1.54), 30–34 (aOR:1.61), and 35–39 (aOR:1.34) were more likely to use a modern contraceptive method during the survey than were women in the age groups of 45–49 (aOR:0.40) [37]. The inclination of women aged 15–24 to use contraceptives may be influenced by their pursuit of education and career goals. In contrast, the reduced contraceptive use among older women may stem from the assumption that they are not at risk of pregnancy [38].

A study in a rural Zambia area showed that several factors influencing modern contraception utilisation were having a child, being married, being older than 19, being visited by a field health worker, being exposed to family planning messages on mass media, and belonging to the wealthiest group [27]. Muanda’s study also stated that inadequate spousal communication, sociocultural norms (particularly the husband’s role as the primary decision-maker and desire for a large family), fear of adverse effects, and a lack of knowledge all contributed to the lack of contraceptive use in rural areas [28]. Overall, in the current study, the trend in urban and rural areas is quite the same: younger people use modern contraceptives more often than older people, which is a positive trend from a public health perspective. However, older women who are considered high-risk if pregnant also need to be supported to use modern contraceptives effectively.

The present study also found that occupation is associated with modern contraceptive use. Married women who were not working had a slightly higher likelihood of modern contraceptive use compared to married women who were working. In contrast to a previous study in Liberia, this study stated that being employed independently is associated with modern contraceptive utilisation [30, 39]. This could be attributed to the fact that employed married women may experience improved financial status, enhanced access to media, and better healthcare services, all of which may contribute positively to the utilisation of modern contraceptives among women [30]. Married women who are unemployed exhibit a higher likelihood of utilizing modern contraceptives than their employed counterparts, potentially attributable to job-related inflexibility that restricts working women’s capacity to take time off during standard hours for midwifery care, including family planning services [40].

Married women with primary education had 4.8 times higher odds of using modern contraceptives in urban areas and 3.9 times higher odds in rural areas compared to those with higher education. This findings are similar to a previous study in Ethiopia indicating women from the wealthiest quintile, with primary education, from the SNNPR/Amhara region, and with 1 to 2 children are more likely to use modern contraceptives compared to women from the poorest quintile, with no education, from Tigray, and with no children, respectively [37]. However, the current study contrasted with a study among sexually active women in Rwanda, which found that between 2010 and 2020, the likelihood of women with secondary education or above increased, which had a considerable favourable impact on the usage of contemporary contraceptives (7.36%) [41]. A prior study indicated that modern contraceptive use exerted a more significant influence on fertility rates than education, although education also contributed to fertility reduction, with its direct impact surpassing its indirect effect via contraceptive use [42].

In line with married women’s education and partner’s education, married women whose partners had primary education had higher odds of using modern contraception than married women whose partners had higher education in both areas. Rahayu’s study also showed that male participation in family planning in Indonesia remains low, with just 8% of men utilising contraception, according to the 2017 IDHS data [43]. Another study on male involvement in family planning in Indonesia found that men with higher education levels showed higher scores in communication, positive gender role perspective, and active direct involvement but not in approval of family planning, reflecting its near-universal acceptance among Indonesian men [43]. This study contrasts with a previous survey in Liberia that found that educated husbands were more likely to use modern contraceptives [30].

Partner’s education is also strongly associated with modern contraceptive use. Previous research indicated that cultural norms influence reproductive behaviour, with the patriarchal culture in Indonesia shaping and encouraging women to conform to traditional roles, thereby limiting their autonomy in decisions regarding family planning methods [44]. Regarding the usage of modern contraceptives, a study using secondary data from the Performance Monitoring and Accountability (PMA) survey among 10,210 women in Indonesia in 2020 showed that women who possess a greater degree of autonomy in making decisions regarding family planning, those who have access to free national or district health insurance, and those residing in communities where community health workers visit a more significant proportion of women, exhibited a higher likelihood of utilising modern contraceptive methods [45]. Although Indonesia’s total fertility rate declined to 2.18 in 2020, ensuring that women have the autonomy to make choices and access family planning and contraceptive services remains essential [46, 47].

Exposure to FP messages on television was not significantly associated with modern contraceptive use in urban or rural areas. In urban areas, the percentage of women who heard FP messages on TV was slightly higher than those who did not, while in rural areas, the percentages were relatively similar. Television is an effective and popular form of mass entertainment, and 86% of Indonesians have television [48]. People might perhaps trust the information provided by television more than other media. The result that FP in TV increased usage of FP was supported by another study in Senegal, in which media exposure was associated with modern contraceptive utilisation [49]. More education on TV may be beneficial, especially in a rural area, with access to correct information about FP on TV and following the recommendation of using modern contraceptives.

In contrast to information on television, married women who have heard about family planning (FP) through the internet have a significantly lower likelihood of modern contraceptive use than those who have not heard about it on the internet (Table 1). This is not in line with the results of research conducted in Ethiopia, where the internet was used to provide information about family planning to increase public knowledge about family planning [50].

Gaining information about family planning for married couples is important, especially for low-income families who have many children [51]. However, this study found that married women who heard FP on the internet were more likely to use modern contraceptives less. This might be because they have already read information about the side effects of modern contraceptives and other alternative contraceptive methods. The internet provides an increasing amount of information on general health, and it has a powerful impact on society [52]. The validation of information is not systematic, and everyone can share their own information. However, comprehensive and validated counseling about contraceptive options, including accurate information about side effects, and the development of new contraceptive technologies are needed for women in low-income countries [53].

Recently, Indonesians have increasingly turned to social media and the internet for health information, with around 40% of fake news articles circulating in Indonesia in 2019 being health-related, according to data from the Indonesian Telecommunication Society (Mastel) [54–56]. If news or information is not filtered thoroughly, this can lead to misinformation about the public acceptance of contraceptives. Rass’ study suggests that to avoid false information, people may try to use multiple and reliable sources of information [57]. In turn, false information leads women not to use modern contraceptives. An evaluation is needed to determine what the family planning program provides through information and communication technology. Access to mobile phones and the internet is an effective way to educate women more comprehensively, attracting their interest in using modern contraceptives [58].

Regarding the number of children, married women with 1–3 children in urban areas had 5.9 times higher odds of using modern contraceptives compared to married women with no children, while married women with 4–6 children in urban areas had 4.1 times higher odds of using modern contraceptives compared to married women with no children. In rural areas, married women with 1–3 children had 2.3 times higher odds of using modern contraceptives than married women without children. The high likelihood of multipara married women who use modern contraception is an important result, as it allows these women to plan their families.

In addition, married women with ≥ 7 children have the lowest likelihood of modern contraceptive use, while married women with 1–3 children have the highest rate in urban. This study precisely contrasts with a previous survey in Liberia that indicated that having many children leads to being more aware of using modern contraceptives, having 1–2 child/children [AOR = 2.81], 3–4 children [AOR = 3.87] and five and above [AOR = 7.06] [30]. Having many children is risky. The clinical application of contraceptive methods guarantees that women or couples select and consistently utilize the suitable method to avert pregnancy [59]. Healthcare practitioners and organizations must address the needs of this demographic, characterized by a high number of children and low utilization of modern contraceptives, as they are at significant risk and likely to have further offspring. Conversely, healthcare providers should recognize and encourage other groups that excel in the utilization of contemporary contraceptives [60, 61].

According to the wealth index variable, poorer urban areas are more likely to use contraception compared to their counterparts, whereas the wealthiest rural areas are more likely to use contraception compared to their counterparts. A study conducted in Benin [62] found that the wealthiest women were more likely to use modern contraceptives than the poorest women. Universally, the majority of studies show that lower socioeconomic status is associated with more access to healthcare barriers compared to wealthy women who can afford both the service itself and the travel costs to the health facility [62, 63].

Furthermore, our study revealed a trend in modern contraceptive use among married women in rural areas, particularly those aged 15–19 and 20–24, women with no education or only primary education, married women whose partners have no education or only primary education, women with 1–3 live births, those with media exposure, and among the wealthiest in rural areas. This was supported by the results of data analysis from the 2019 Program Performance and Accountability Survey for West Java, Indonesia, that showed sociodemographic factors related to contraceptive use were younger age, higher education level, lower wealth quintile, and living in rural [64]. Another group who are less adept at using modern contraceptives implies that, to narrow the gap in adopting modern contraceptives, the government and healthcare providers need to allocate greater focus to improve women’s decision-making regarding family planning use [65]. Moreover, media exposure should be emphasised more in campaigns and provide more positive knowledge about family planning.

In this case, the modest urban-rural disparity in Indonesia can be ascribed to the execution of comprehensive family planning initiatives, bolstered by the National Health Insurance (JKN), which seeks to improve access to contraceptive services throughout all regions, including rural locales [66]. Family planning is crucial for controlling population growth and reducing maternal mortality rates (MMR) in Indonesia. Modern contraception, as a key public health intervention, not only helps prevent unwanted pregnancies and reduces maternal mortality but also offers broader benefits, such as empowering women, increasing educational opportunities, and promoting sustainable population growth and economic development, particularly in developing countries where usage remains low among reproductive-aged women [67, 68].

**Fig. 1:**
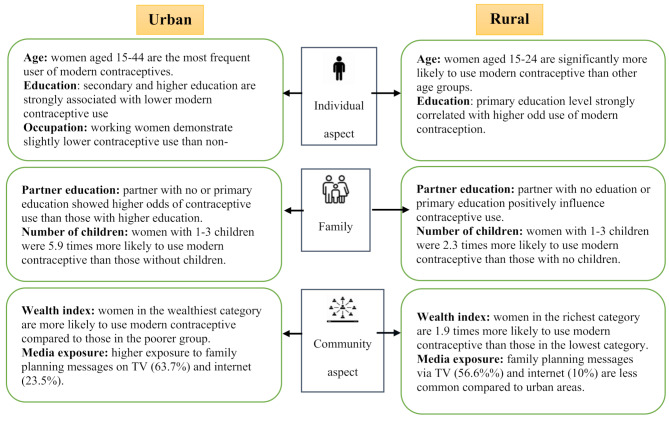
Determinants of rural-urban utilisation of modern contraceptives among married women in Indonesia

In a nutshell, the results of this study are shown in Fig. 1 below.

### The limitation of the study

This cross-sectional study cannot determine causality between variables. It employed extensive, globally standardized survey data. The study’s limitations encompass possible memory bias from self-reported contraceptive usage and the inability to comprehensively account for regional or cultural variations in contraceptive access and utilization. Recognizing these constraints and citing analogous studies might enhance the contextual clarity for interpreting the results. Despite the limitations, this study provides novel insight into the determinants of modern contraceptive use in Western Indonesia, assisting in designing future programs to increase modern contraceptive use both in urban and rural areas.

## Conclusion

The rate of modern contraceptive use in urban and rural areas of Western Indonesia remains consistently similar. This analysis found that sociodemographic factors are significantly associated with contraceptive use. The key difference between the two settings lies in the varying influence of family planning factors such as education level, number of children, and wealth index. Modern contraceptive use is lower among women with secondary education, indicating a need for targeted education and counseling to address potential knowledge gaps or misconceptions. In urban areas, women with 1–3 children are more likely to use modern contraceptives, suggesting that family planning promotion should begin early in the reproductive life cycle. In rural areas, women in the highest wealth group have higher contraceptive use than those in the lowest group, highlighting the importance of expanding free or subsidized services for the lowest wealth group. Conversely, in urban areas, women in the lowest wealth group are more likely to use contraceptives than those in the highest group, suggesting that outreach and education efforts should also be strengthened for higher-income women through health facilities and digital platforms.

## Data Availability

All data generated in this study are included in this manuscript.

## Abbreviations

FP: Family planning
MMR: Maternal mortality ratio
ANC: Antenatal care
SDG: Sustainable Development Goals
TBA: Traditional birth attendants
IUD: Intrauterine devices
CPR: Contraceptive prevalence rate
IDHS: Indonesian Demographic and Health Survey
ICF: Inner-City Fund
OCR: Office of Client Resources
LAM: Lactation amenorrhea method
SDM: Standard days method
OR: Odds ratio
CI: Confidence interval
TV: Television
DHS: Demographic and Health Survey
DRC: Democratic Republic of Congo
ZDHS: Zambia Demographic and Health Survey
PMA: Performance Monitoring and Accountability
SNNPR: Southern Nations and Nationalities Peoples Region
